# Genetic Evidence for Repurposing of GLP1R (Glucagon‐Like Peptide‐1 Receptor) Agonists to Prevent Heart Failure

**DOI:** 10.1161/JAHA.120.020331

**Published:** 2021-06-29

**Authors:** Iyas Daghlas, Ville Karhunen, Devleena Ray, Verena Zuber, Stephen Burgess, Philip S. Tsao, Julie A. Lynch, Kyung Min Lee, Benjamin F. Voight, Kyong‐Mi Chang, Emma H. Baker, Scott M. Damrauer, Joanna M. M. Howson, Marijana Vujkovic, Dipender Gill

**Affiliations:** ^1^ Harvard Medical School Boston MA; ^2^ Department of Epidemiology and Biostatistics School of Public Health Imperial College London London UK; ^3^ Medical Research Council Biostatistics Unit Cambridge Institute of Public Health Cambridge UK; ^4^ Department of Public Health and Primary Care University of Cambridge Cambridge UK; ^5^ VA Palo Alto Health Care System Palo Alto CA; ^6^ Department of Medicine Stanford University School of Medicine Stanford CA; ^7^ Department of Veterans Affairs VA Informatics and Computing Infrastructure Salt Lake City Health Care System Salt Lake City UT; ^8^ Department of Internal Medicine University of Utah School of Medicine Salt Lake City UT; ^9^ Corporal Michael J. Crescenz VA Medical Center Philadelphia PA; ^10^ Department of Genetics University of Pennsylvania Perelman School of Medicine Philadelphia PA; ^11^ Department of Systems Pharmacology and Translational Therapeutics University of Pennsylvania Perelman School of Medicine Philadelphia PA; ^12^ Institute of Translational Medicine and Therapeutics University of Pennsylvania Perelman School of Medicine Philadelphia PA; ^13^ Department of Medicine University of Pennsylvania Perelman School of Medicine Philadelphia PA; ^14^ Clinical Pharmacology and Therapeutics Section Institute of Medical and Biomedical Education and Institute for Infection and Immunity St George's University of London London UK; ^15^ Clinical Pharmacology Group, Pharmacy and Medicines Directorate St George's University Hospitals NHS Foundation Trust London UK; ^16^ Department of Surgery Perelman School of Medicine University of Pennsylvania Philadelphia PA; ^17^ Novo Nordisk Research Centre Oxford Oxford UK

**Keywords:** diabetes mellitus, ejection fraction, GLP1R, heart failure, Mendelian randomization, Genetic, Association Studies, Genetics, Heart Failure

## Abstract

**Background:**

This study was designed to investigate the genetic evidence for repurposing of GLP1R (glucagon‐like peptide‐1 receptor) agonists to prevent heart failure (HF) and whether the potential benefit exceeds the benefit conferred by more general glycemic control.

**Methods and Results:**

We applied 2‐sample Mendelian randomization of genetically proxied GLP1R agonism on HF as the main outcome and left ventricular ejection fraction as the secondary outcome. The associations were compared with those of general glycemic control on the same outcomes. Genetic associations were obtained from genome‐wide association study summary statistics of type 2 diabetes mellitus (228 499 cases and 1 178 783 controls), glycated hemoglobin (n=344 182), HF (47,309 cases and 930 014 controls), and left ventricular ejection fraction (n=16 923). Genetic proxies for GLP1R agonism associated with reduced risk of HF (odds ratio per 1 mmol/mol decrease in glycated hemoglobin 0.75; 95% CI, 0.64–0.87; *P*=1.69×10^−4^), and higher left ventricular ejection fraction (SD change in left ventricular ejection fraction per 1 mmol/mol decrease in glycated hemoglobin 0.22%; 95% CI, 0.03–0.42; *P*=0.03). The magnitude of these benefits exceeded those expected from improved glycemic control more generally. The results were similar in sensitivity analyses, and we did not find evidence to suggest that these associations were mediated by reduced coronary artery disease risk.

**Conclusions:**

This genetic evidence supports the repurposing of GLP1R agonists for preventing HF.

Patients with type 2 diabetes mellitus are at increased risk of developing heart failure and evidence from randomized controlled trials supports that GLP1R (glucagon‐like peptide‐1 receptor) agonists reduce this risk.^1^, ^2^ The aim of this study was to leverage human genetic data within the Mendelian randomization paradigm to investigate whether effects of GLP1R agonists on heart failure risk and left ventricular ejection fraction (LVEF) exceed those of improved glycemic control more generally.

## METHODS

All data used in this work are publicly available and anonymized. All contributing studies received appropriate ethical approval and patient consent.

### Methodologic Overview

The Mendelian randomization (MR) approach uses genetic variants as proxies to investigate the causal effect of an exposure on an outcome.^3^, ^4^ This method leverages the random allocation of genetic variants at conception to reduce any bias due to confounding and reverse causation that can limit causal inference in observational research. MR can be extended to investigate drug effects by leveraging genetic variation in genes (eg, *GLP1R*) encoding proteins corresponding to drug targets.^5^

### Genetic Proxies for GLP1R Agonism and Glycemic Control

We identified genetic proxies for the effect of GLP1R agonism as genome‐wide significant (*P*<5×10^−8^) and uncorrelated (*r* ^2^<0.1) variants in the *GLP1R* gene (genomic position on build GRCh37/hg19: chromosome 6:39 016 574–39 055 519) that associated with type 2 diabetes mellitus liability in the largest published genome‐wide association study meta‐analysis (228 499 cases and 1 178 783 controls; 79% European ancestry),^6^ with directionally concordant and nominally significant (*P*<0.05) associations with glycated hemoglobin in the UK Biobank (n=344 182).^7^ Unless otherwise stated, all downstream analyses were weighted by the variant association with glycated hemoglobin (mmol/mol). These variants were annotated for their sequence effects (eg, intron or missense), and we queried the Genotype‐Tissue Expression v8 data set of 54 tissue types to determine whether the variants were associated with gene expression.^8^ Variants were annotated as having directionally concordant associations with gene expression if they were associated with lower glycated hemoglobin and greater expression of *GLP1R* (or vice versa).

Genetic proxies for glycemic control more generally were identified through the same associations but considering genetic variants throughout the genome that were not located within 1megabase of *GLP1R*. Given the larger number of variants identified from throughout the genome, we used a stricter clumping threshold of *r* ^2^<0.001 to minimize bias due to linkage disequilibrium.

### Heart Failure and Left Ventricular Ejection Fraction Genetic Association Estimates

Heart failure was the primary outcome for our analysis. We obtained genetic association estimates from the Heart Failure Molecular Epidemiology for Therapeutic Targets Consortium consisting of 47 309 cases and 930 014 controls of European ancestry.^9^ Cases included patients with a clinical diagnosis of heart failure, irrespective of the ejection fraction. We further investigated LVEF as a secondary outcome using genetic association estimates from a study of cardiac magnetic resonance imaging derived LVEF in the UK Biobank (n=16 923, all of European ancestry).^10^ LVEF was inverse normal‐transformed, and the genetic association estimates are therefore presented in approximate SD units.

### Statistical Analysis

For each of the variants used in MR analysis, we harmonized genetic associations with the exposure and outcome by aligning effect alleles, with no exclusion made for palindromic variants. We derived MR estimates considering genetically proxied GLP1R agonism and glycemic control more generally using the random‐effects inverse‐variance weighted method with intercept fixed at the origin,^3^ orientating estimates to reduction in glycated hemoglobin (ie, the direction of drug effect). All MR analyses were performed using the TwoSampleMR package in R.^3^ To assess for a GLP1R agonism drug class effect that exceeds the anticipated effect of glycemic control more generally, we tested for a significant difference between the respective MR estimates. The point estimate for this difference was obtained by taking the difference between the MR beta coefficients for the GLP1R and glycemic control estimates, and the SE for the difference was derived using the propagation of error method:

$$SE\left(\beta_{GLP1R}‐\beta_{\mathrm{GLYCEMIA}}\right)=\sqrt{SE{\left(\beta_{GLP1R}\right)}^{2}+SE{\left(\beta_{\mathrm{GLYCEMIA}}\right)}^{2}},$$

where $\beta_{GLP1R}$ and $\beta_{\mathrm{GLYCEMIA}}$ are the MR estimates for the associations of genetically proxied GLP1R agonism and glycemic control with the outcomes.

Analyses investigating LVEF as a secondary outcome were considered exploratory, and so the *P* values were not corrected for multiple comparisons. All hypothesis tests were 2 sided.

### Sensitivity Analyses

In sensitivity analyses considering GLP1R agonism we restricted the genetic proxies to coding variation in *GLP1R*, as these variants more plausibly relate to *GLP1R* function. Corresponding MR estimates that used a single proxy variant were derived using the Wald ratio with first‐order SEs. We also performed analyses excluding any coding variants to ensure that they were not solely driving the MR estimates. To determine whether results were sensitive to our choice to weight the variants by their associations with glycated hemoglobin, we also performed analyses weighted by the log‐odds of type 2 diabetes mellitus liability. MR estimates may be biased by horizontal pleiotropy if the genetic variants proxying GLP1R agonism influence heart failure risk or LVEF through a pathway independent of GLP1R agonism. We first tested for any such bias by calculating the Cochran Q test *P* value to assess for overdispersion in the MR estimates provided by each variant in the GLP1R agonism instrument. We then performed analyses using the weighted median method, which provides consistent MR estimates if more than half of the weight from the genetic proxies comes from valid instrumental variables.^11^

To determine whether protective effects of GLP1R agonism on heart failure may be mediated by reduced coronary artery disease risk, we performed MR analyses investigating the effect of GLP1R agonism on coronary artery disease risk. We obtained genetic association estimates from a meta‐analysis of data from the CARDIOGRAMplusC4D Consortium and UK Biobank consisting of 122 733 cases and 424 528 controls of European ancestry.^12^

## RESULTS

### Identification of Genetic Proxies for GLP1R Agonism and Glycemic Control More Generally

Three independent variants in *GLP1R* were identified as genetic proxies for GLP1R agonism, including 1 missense variant (rs10305420) and 2 intronic variants (rs2268647 and rs75151020; Tables S1–S2). Two of these variants were significantly associated with expression of *GLP1R* across several human tissues, and both variants had directionally concordant associations with *GLP1R* expression in pancreatic tissue (Table S3). A directionally discordant association with *GLP1R* expression in left ventricular and left atrial appendage myocardial tissue was identified for the intronic variant rs2268647 (Table S3). There were 350 variants available for use as proxies for glycemic control more generally in the heart failure data set (Table S4) and 334 variants available in the LVEF data set (Table S5).

### Mendelian Randomization Analyses

Genetically proxied GLP1R agonism associated with a reduced risk of heart failure (odds ratio [OR] per 1 mmol/mol decrease in glycated hemoglobin, 0.75; 95% CI, 0.64–0.87; *P*=1.69×10^−4^). This estimate was similar in MR analysis only using the missense variant rs10305420 (OR, 0.62; 95% CI, 0.45–0.85; *P*=2.59×10^−3^). Analyses excluding this variant provided similar evidence of effect, suggesting that this variant did not solely drive the estimates (OR, 0.79; 95% CI, 0.67–0.92; *P*=3.33×10^−3^). Consistent with previous reports,^9^ a genetically proxied improvement in overall glycemic control associated with reduced risk of heart failure (OR, 0.96; 95% CI, 0.94–0.97; *P*=7.75×10^−11^). This estimate was smaller in magnitude than the estimate obtained for genetically proxied GLP1R agonism (*P*_difference_=1.58×10^−3^; Figure).

Genetically proxied GLP1R agonism associated with a higher LVEF (SD change in LVEF, 0.22; 95% CI, 0.03–0.42; *P*=0.03). There was no evidence of an association between genetically proxied glycemic control more generally and LVEF (SD change in LVEF, 0.00; 95% CI, −0.01 to 0.02; *P*=0.67). This estimate was smaller in magnitude than that obtained for genetically proxied GLP1R agonism (*P*_difference_=0.03). Corresponding scatter plots for all analyses are provided in Figures S1–S5.

### Sensitivity Analyses

Analyses weighting the genetic proxies for GLP1R agonism (OR per log‐odds increase in type 2 diabetes mellitus liability, 0.48; 95% CI, 0.34–0.67; *P*=2.87×10^−5^) and overall glycemic control (OR, 0.90; 95% CI, 0.87–0.93; *P*=1.81×10^−12^) by type 2 diabetes mellitus liability showed similar evidence for a reduction in heart failure risk. There was no significant heterogeneity in the MR estimates generated by the different variants when considering either heart failure or LVEF as outcomes (Figures S1–S3). Results from analyses using the weighted median method showed significant protective associations of genetically proxied GLP1R agonism (OR, 0.77; 95% CI, 0.62–0.96; *P*=0.02) and improved glycemic control (OR, 0.98; 95% CI, 0.96–1.00; *P=*0.04) with heart failure risk, and directionally concordant but nonsignificant associations of genetically proxied GLP1R agonism with LVEF (SD change in LVEF, 0.18; 95% CI, −0.07 to 0.42; *P*=0.16; Table S6). We found no evidence for an association of genetically proxied GLP1R agonism with coronary artery disease risk (OR per 1 mmol/mol decrease in glycated hemoglobin, 1.02; 95% CI, 0.89–1.16; *P*=0.80).

## DISCUSSION

In this MR study, we used human genetic data to identify proxies for GLP1R agonism and found evidence for their protective effect on risk of heart failure. In secondary analyses, we found associations of genetically proxied GLP1R agonism with increased LVEF. The magnitude of these estimates exceeded those generated using genetic proxies for glycemic control more generally, supporting a role for GLP1R signaling in preventing heart failure beyond an effect on glycemic control alone.^13^ We did not find evidence of heterogeneity in the MR estimates generated by the genetic proxies for GLP1R agonism when considering either heart failure or LVEF as outcomes, and results were similar in sensitivity analyses using the weighted median method, with the null effect of GLP1R agonism on LVEF potentially attributable to low statistical power. The null effect of genetically proxied GLP1R agonism on coronary artery disease risk suggests that a reduced risk of heart failure is not attributable to chronic ischemic heart disease.

Our findings are consistent with meta‐analyses of randomized controlled trials identifying a protective effect of GLP1R agonists on hospital admission with heart failure^1^, ^2^ and go further to provide genetic evidence supporting a drug effect on LVEF. Further clinical research is needed to determine contexts where GLP1R agonists may be repurposed for reducing risk of heart failure, particularly given the established effects of sodium glucose cotransporter 2 inhibitors for reducing progression of heart failure in patients with and without type 2 diabetes mellitus.^14^

A similar genetic approach was previously used to support a protective effect of GLP1R agonism on coronary artery disease risk^15^; however, our analyses did not replicate this finding. The previous investigation used a low‐frequency missense variant, rs10305492, which is not in strong linkage disequilibrium with any of the variants included in our investigation (all pairwise r^2^<0.16). We did not select this variant for inclusion in our analysis as its association with type 2 diabetes mellitus achieved only a nominal level of statistical significance (*P*=0.001), and not the more stringent genome‐wide level of statistical significance achieved by the variants in our investigation. In contrast, meta‐analyses of clinical trials have supported a nominally significant (*P*=0.043 before adjustment for multiple comparisons) beneficial effect of GLP1R agonism on myocardial infarction.^1^ Given the small magnitude of this reported effect (hazard ratio, 0.91; 95% CI, 0.84–1.00^1^) and the span of the CIs from our MR estimates (95% CI, 0.89–1.16), it is plausible that the null MR estimate for coronary artery disease is attributable to low statistical power.

The key strength of our work is the use of randomly allocated genetic proxies to study the effects of GLP1R agonism. The genetic proxies used in these analyses were further validated by their associations with glycated hemoglobin, which reduces risk of bias due to winner's curse and permits the contextualization of the MR estimates on the glycated hemoglobin scale. The genetic associations with LVEF were adjusted for body mass index, which allowed standardization for body size. A study limitation is the absence of available large‐scale genetic summary data for heart failure subtypes. Although we used a missense variant in *GLP1R* (rs10305420) and gene expression data to strengthen the validity of our findings, further experimental work is necessary to determine the mechanism by which these variants influence *GLP1R* expression or function. In particular, the directionally concordant association of the rs2268647 intronic variant on gene expression in the pancreas, but discordant effect on *GLP1R* expression in myocardial tissue warrants further exploration. The MR estimates reflect the consequence of a lifelong genetic perturbation of GLP1R signaling and cannot be extrapolated to predict the magnitude of effect from shorter, discrete pharmacological interventions. The limited number of genetic variants available to instrument GLP1R agonism precluded more extensive sensitivity analyses for horizontal pleiotropy. In particular, modeling an intercept term (as in the MR‐Egger regression approach) can in some scenarios mitigate bias from unbalanced horizontal pleiotropy but was not appropriate in our analysis of GLP1R agonism because of the availability of only 3 genetic proxies. We used summary‐level genetic associations with heart failure and LVEF and therefore could not perform stratified analyses, such as by sex or diabetes mellitus status. Finally, these genetic data were predominantly gathered from individuals of European ancestry and these results may therefore not generalize to other ethnic groups.

## CONCLUSIONS

In conclusion, we identified genetic proxies for the effects of GLP1R agonism, and applied these proxies in MR analyses to generate evidence supporting a protective effect on risk of heart failure. Further investigation of GLP1R agonist repurposing to prevent heart failure in the context of clinical trials is warranted.

## Sources of Funding

D.G. was supported by the Wellcome Trust 4i Programme (203928/Z/16/Z) and British Heart Foundation Centre of Research Excellence (RE/18/4/34215) at Imperial College, and a National Institute for Health Research Clinical Lectureship (CL‐2020‐16‐001) at St. George's, University of London. S.B. is supported by a Sir Henry Dale Fellowship jointly funded by the Wellcome Trust and the Royal Society (204623/Z/16/Z). B.F.V. was supported by the National Institutes of Health (DK101478) and a Linda Pechenik Montague Investigator award. P.S.T. and K.‐M.C. are supported by the VA Cooperative Studies Program with funding from the VA award I01‐BX003362. S.M.D. was supported by the Department of Veterans Affairs Office of Research and Development (IK2‐CX001780). Million Veterans Program research data are funded by Office of Research and Development, Veterans Health Administration and supported by award no. MVP000. MVP‐based articles do not represent the views of the VA, the US Food and Drug Administration, or the US Government. This work was supported by funding from the National Institute for Health Research (NIHR; Cambridge Biomedical Research Centre at the Cambridge University Hospitals National Health Service [NHS] Foundation Trust). The views expressed are those of the authors and not necessarily those of the NHS, the NIHR or the Department of Health and Social Care. The funding sources had no role in the design, acquisition of data, analysis, interpretation or write up of this study.

## Disclosures

D.G. is employed part time by Novo Nordisk and has received consultancy fees from Policy Wisdom. J.M.M.H. is an employee of Novo Nordisk. S.M.D. has received grants from the U.S. Department of Veterans Affairs, Calico Labs, and Renalytix AI plc outside the submitted work. The remaining authors have no disclosures to report.

**Figure 1:**
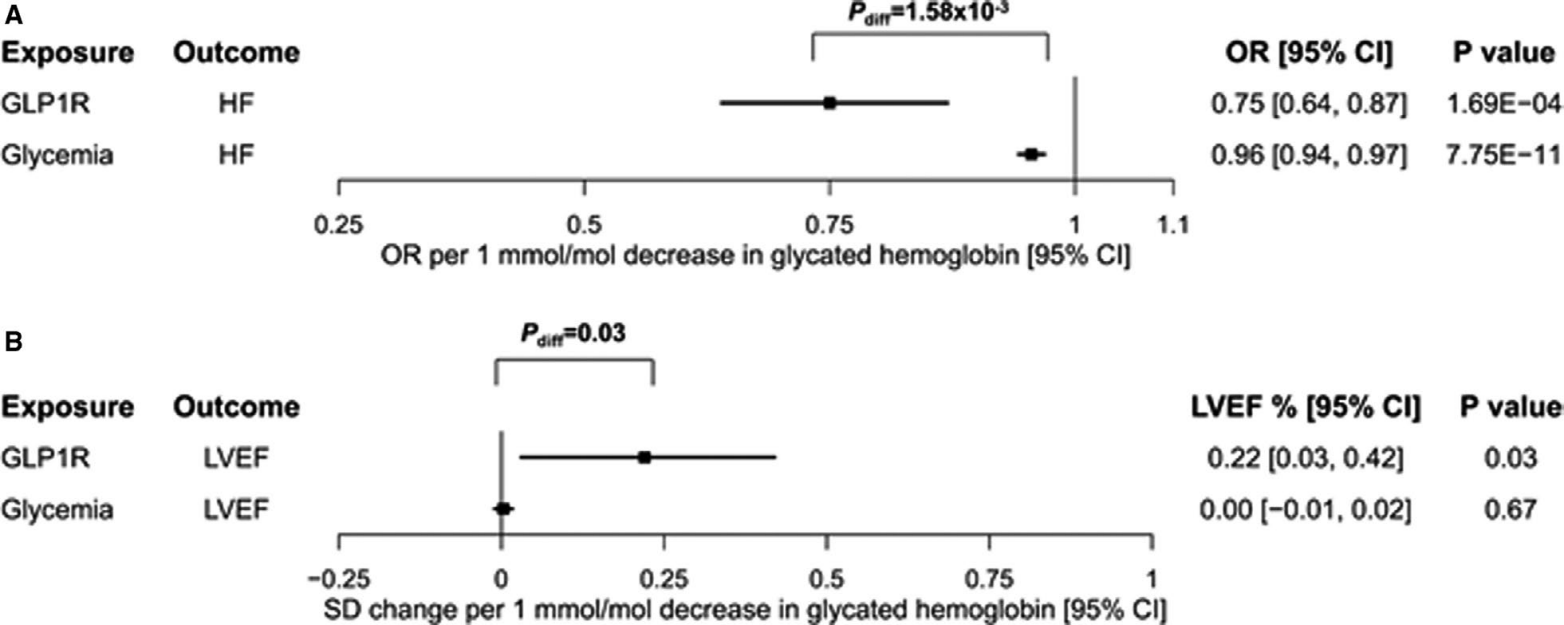
Forest plot depicting Mendelian randomization estimates for the association of genetically proxied GLP1R (glucagon‐like peptide receptor) agonism and glycemic control more generally with (**A**) risk of heart failure (HF; 47 309 cases/930 014 controls) and (**B**) left ventricular ejection fraction (LVEF; n=16 923). Estimates reflect the effect of a reduction in glycated hemoglobin on each of the respective outcomes (so as to orient estimates to GLP1R agonist drug effects). Squares correspond to point estimates, and the surrounding lines correspond to 95% CIs. diff indicates difference; and OR, odds ratio.

## Supporting information

Tables S1–S6
Figures S1–S5
