# Supplementary material for: Genetic Evidence for Repurposing of GLP1R (Glucagon‐Like Peptide‐1 Receptor) Agonists to Prevent Heart Failure
Source: J Am Heart Assoc. 2021 Jun 29;10(13):e020331. doi: 10.1161/JAHA.120.020331 (PMC8403330; doi:10.1161/JAHA.120.020331)
Supplement: Supplementary file 1 — Tables S1–S6 Figures S1–S5 [file JAH3-10-e020331-s001.pdf]

SUPPLEMENTAL MATERIAL

Table S1. Genetic proxies for GLP1R agonism, and estimates for their association with glycated hemoglobin, type 2 diabetes, heart failure, and left ventricular ejection fraction.

						Glycated hemoglobin			Type 2 diabetes			Heart failure			Left ventricular ejection fraction		
**SNP**	**Chr**	**Position**	**EA**	**OA**	**EAF**	**Beta**	**SE**	**P**	**Beta**	**SE**	**P**	**Beta**	**SE**	**P**	**Beta**	**SE**	**P**
rs10305420*	6	39016636	T	C	0.39	-0.051	0.016	1.30×10^{-3}	-0.032	0.004	5.11×10^{-14}	-0.024	0.008	2.63×10^{-3}	-0.009	0.011	4.1×10^{-1}
rs75151020	6	39031592	C	A	0.09	0.119	0.026	7.08×10^{-6}	0.041	0.007	1.37×10^{-9}	-0.022	0.013	1.04×10^{-1}	0.040	0.019	3.4×10^{-2}
rs2268647	6	39043178	T	C	0.52	0.066	0.015	1.51×10^{-5}	0.021	0.004	4.95×10^{-8}	0.019	0.007	1.12×10^{-2}	0.008	0.011	4.4×10^{-1}

Chr: chromosome; EA: effect allele; EAF: effect allele frequency; OA: other allele; SE: standard error; SNP: single nucleotide polymorphism. *: missense variant

Table S2. Linkage disequilibrium r^2 values for variants used as proxies for GLP1R agonism.

rsID	rs10305420	rs75151020	rs2268647
**rs10305420**	1.00	0.01	0.00
**rs75151020**	0.01	1.00	0.07
**rs2268647**	0.00	0.06	1.00

r^2 values were obtained using linkage disequlibrium data from the European subsample of the 1000 Genomes project (https://ldlink.nci.nih.gov/?tab=ldmatrix).

Table S3. Genetic proxies for GLP1R agonism, and estimates for their association with gene expression in the GTEx v8 database.

Gene Symbol	SNP	P-Value	NES	Directional concordance with glycated hemoglobin	Tissue
GLP1R	rs10305420	2.10E-09	0.23	Yes	Nerve - Tibial
GLP1R	rs10305420	3.80E-06	0.26	Yes	Adipose - Visceral (Omentum)
GLP1R	rs10305420	4.10E-06	0.25	Yes	Thyroid
GLP1R	rs10305420	2.30E-05	0.24	Yes	Pancreas
GLP1R	rs2268647	1.00E-09	0.31	No	Heart - Left Ventricle
GLP1R	rs2268647	2.10E-07	0.23	No	Heart - Atrial Appendage
GLP1R	rs2268647	3.40E-07	-0.33	Yes	Stomach
GLP1R	rs2268647	2.10E-06	-0.27	Yes	Pancreas
GLP1R	rs2268647	1.60E-05	-0.23	Yes	Thyroid
ANKRD18EP	rs2268647	2.20E-05	-0.12	Yes	Skin - Sun Exposed (Lower leg)

rs75151020 did not significantly influence gene expression in any of the tissues in the GTEx database. Variants were annotated as directionally concordant if they were associated with lower glycated hemoglobin and higher expression of *GLP1R* (or vice versa). NES: normalized effect size; SNP: single nucleotide polymorphism.

Table S4. Genetic proxies for glycemic control by any mechanism, and estimates for their association with glycated hemoglobin (for the outcome of heart failure).

SNP	Chromosome	Position	Effect allele	Other allele	EAF	Beta	SE
rs2482506	10	104563743	G	C	0.25	-0.053	0.018
rs79364741	10	114666651	T	C	0.01	-0.164	0.073
rs11196174	10	114734096	G	A	0.29	0.252	0.017
rs149692182	10	114752674	T	C	0.02	0.309	0.053
rs35676242	10	114757314	A	C	0.05	0.230	0.036
rs11257655	10	12307894	T	C	0.21	0.219	0.019
rs946859	10	13565429	A	G	0.47	-0.075	0.015
rs3122231	10	44027356	C	T	0.65	0.050	0.016
rs113899647	10	64850074	T	C	0.03	-0.189	0.044
rs949693	10	70354574	A	G	0.61	-0.050	0.016
rs11592899	10	71333783	A	G	0.34	-0.055	0.016
rs2812535	10	71456857	A	G	0.62	0.069	0.016
rs697239	10	80947438	C	T	0.46	-0.105	0.015
rs11201992	10	88117318	A	C	0.46	-0.038	0.015
rs1111875	10	94462882	T	C	0.41	-0.181	0.016
rs66536955	10	94737667	C	T	0.26	0.044	0.017
rs34041345	10	99174580	G	T	0.26	0.060	0.018
rs529623	11	117693255	C	T	0.52	-0.059	0.015
rs10893830	11	128044159	T	C	0.13	-0.058	0.023
rs10750397	11	128234144	G	A	0.72	-0.040	0.017
rs67232546	11	128398938	T	C	0.21	0.067	0.019
rs117316450	11	14518419	G	C	0.02	0.316	0.054
rs757110	11	17418477	A	C	0.64	-0.112	0.016
rs11042987	11	2201059	A	C	0.58	-0.034	0.016
rs10831668	11	2288412	T	C	0.02	0.234	0.060
rs231362	11	2691471	G	A	0.52	0.120	0.015
rs10767659	11	27686196	T	G	0.67	-0.041	0.016

rs60808706	11	2857233	A	G	0.05	-0.227	0.035
rs2289488	11	2892955	C	G	0.40	0.040	0.016
rs62618693	11	32956492	T	C	0.05	-0.144	0.037
rs523472	11	35031668	A	G	0.72	-0.056	0.017
rs3816605	11	47857253	C	T	0.45	-0.080	0.015
rs7483027	11	58128015	C	T	0.38	-0.061	0.016
rs174541	11	61565908	C	T	0.36	-0.098	0.016
rs1143756	11	65299595	G	A	0.29	0.100	0.017
rs3918296	11	69459036	G	C	0.03	-0.249	0.049
rs11602873	11	72460762	T	A	0.16	-0.187	0.021
rs11236524	11	75464344	C	T	0.09	0.069	0.027
rs4945090	11	76205018	A	T	0.60	0.036	0.016
rs12802861	11	8387806	T	C	0.28	-0.052	0.017
rs10830963	11	92708710	G	C	0.28	0.297	0.017
rs3020069	11	93057087	A	G	0.68	0.093	0.016
rs1426371	12	108629780	A	G	0.26	-0.074	0.018
rs79310463	12	118406696	T	C	0.13	0.104	0.023
rs56348580	12	121432117	C	G	0.31	-0.037	0.017
rs7975763	12	123604053	T	C	0.20	-0.057	0.019
rs11614914	12	133070294	T	C	0.33	0.078	0.016
rs12828318	12	133766122	G	A	0.18	-0.057	0.020
rs10841886	12	21864377	C	T	0.23	-0.082	0.018
rs1480029	12	26356032	A	G	0.46	0.042	0.015
rs3751239	12	27963676	G	C	0.20	-0.160	0.019
rs11063018	12	4288001	C	T	0.17	0.067	0.020
rs74862545	12	4365572	T	C	0.02	-0.279	0.052
rs76895963	12	4384844	G	T	0.02	-1.037	0.059
rs2732469	12	48712932	A	T	0.43	-0.258	0.015
rs61937817	12	57212823	G	T	0.11	0.060	0.024
rs11173646	12	61250814	T	A	0.82	-0.046	0.020
rs2257883	12	66216162	A	G	0.13	0.150	0.023
rs12371967	12	66346714	C	T	0.17	-0.043	0.020

rs10879261	12	71520761	G	T	0.41	0.068	0.016
rs11108094	12	95928113	A	C	0.07	0.099	0.030
rs6538805	12	97849120	C	T	0.39	-0.076	0.016
rs9587811	13	109946882	A	C	0.41	-0.056	0.016
rs314879	13	23309382	T	C	0.79	-0.069	0.019
rs34584161	13	26776999	G	A	0.24	-0.063	0.018
rs380854	13	33574631	A	G	0.58	-0.058	0.016
rs9316500	13	51094114	G	T	0.29	-0.067	0.017
rs7991679	13	58691107	A	T	0.16	-0.081	0.021
rs1215451	13	80715893	A	G	0.29	-0.131	0.017
rs112324411	14	101258584	T	C	0.07	-0.101	0.032
rs2295388	14	101309759	A	G	0.22	-0.073	0.019
rs4906272	14	103376031	T	C	0.16	0.046	0.021
rs12883788	14	33303540	T	C	0.46	0.060	0.015
rs7147483	14	38804675	C	T	0.25	-0.158	0.018
rs723355	14	47304091	A	G	0.50	-0.034	0.015
rs4902002	14	61229411	A	G	0.71	-0.034	0.017
rs242105	14	69459229	C	A	0.28	0.062	0.017
rs7156625	14	79942647	A	G	0.22	0.037	0.019
rs8010382	14	91963722	G	A	0.41	0.046	0.016
rs8043085	15	38828140	T	G	0.23	0.071	0.018
rs11856877	15	40620560	G	A	0.11	0.071	0.024
rs1473781	15	41818917	A	G	0.35	0.067	0.016
rs149336329	15	52587740	T	G	0.05	-0.272	0.037
rs7163757	15	62391608	T	C	0.43	-0.042	0.015
rs7178762	15	63871292	T	C	0.55	-0.057	0.015
rs9479	15	74328576	G	A	0.49	0.052	0.015
rs8033589	15	75596685	A	G	0.76	0.058	0.018
rs12910361	15	77782335	G	A	0.71	0.161	0.017
rs893617	15	90381278	T	C	0.72	-0.136	0.017
rs2290202	15	91512267	T	G	0.13	0.085	0.023
rs9927842	16	15153717	C	T	0.84	-0.056	0.021

rs8056890	16	28897452	A	G	0.36	0.105	0.016
rs8054556	16	29958216	A	G	0.47	0.077	0.015
rs55857387	16	300388	C	T	0.20	-0.142	0.019
rs8061528	16	3656482	T	C	0.21	0.092	0.019
rs2024449	16	53494617	C	T	0.44	-0.056	0.015
rs1421085	16	53800954	C	T	0.40	0.154	0.016
rs56125990	16	69742387	G	A	0.15	0.065	0.021
rs4788815	16	71634811	T	A	0.66	0.056	0.016
rs72802365	16	75246035	C	G	0.08	-0.163	0.029
rs2966117	16	81599271	T	G	0.48	0.059	0.015
rs11117364	16	88132199	G	A	0.68	0.066	0.017
rs9937296	16	88554480	C	T	0.86	0.069	0.023
rs66461358	16	89535257	C	T	0.15	0.060	0.021
rs12934854	16	950028	A	G	0.17	0.043	0.020
rs925095	17	17344653	T	C	0.39	-0.075	0.016
rs2297508	17	17715317	G	C	0.65	-0.150	0.016
rs117642733	17	21284910	T	C	0.05	0.107	0.039
rs9913225	17	27570622	A	G	0.58	-0.075	0.016
rs1109442	17	34862220	C	T	0.47	0.071	0.015
rs3110641	17	36047417	G	A	0.78	0.091	0.019
rs11651755	17	36099840	T	C	0.51	-0.124	0.015
rs3786017	17	3830340	C	T	0.11	0.054	0.025
rs8071043	17	3988451	C	T	0.33	0.066	0.016
rs1905339	17	40582296	C	T	0.34	0.097	0.016
rs35895680	17	47060322	A	C	0.33	-0.089	0.016
rs366577	17	4854480	T	C	0.60	-0.051	0.016
rs57767539	17	62203059	A	G	0.07	0.136	0.031
rs11658220	17	65646092	A	G	0.10	0.100	0.025
rs12603589	17	65825248	C	T	0.19	0.103	0.020
rs7224711	17	76772288	T	C	0.53	-0.069	0.015
rs303760	18	21083738	T	C	0.35	0.052	0.016
rs16965062	18	31581247	T	C	0.43	0.034	0.015

rs7227272	18	36746623	A	G	0.10	-0.062	0.026
rs410150	18	40066006	T	C	0.80	-0.047	0.019
rs17596995	18	53166594	A	G	0.20	-0.049	0.019
rs1517037	18	56878274	T	C	0.19	-0.093	0.020
rs6567160	18	57829135	C	T	0.23	0.097	0.018
rs74625348	18	60846430	C	G	0.23	-0.044	0.019
rs12963820	18	63426213	A	T	0.27	0.034	0.017
rs7240767	18	7070642	C	T	0.39	0.063	0.016
rs6565922	18	74558999	T	C	0.38	0.078	0.016
rs9384	19	13010643	T	G	0.38	-0.107	0.016
rs10404726	19	18834514	T	C	0.47	-0.035	0.015
rs58542926	19	19379549	T	C	0.08	0.139	0.029
rs924150	19	31829903	C	A	0.39	-0.087	0.016
rs4805881	19	33896432	C	A	0.67	-0.077	0.016
rs429358	19	45411941	C	T	0.16	-0.142	0.021
rs8107527	19	46158417	A	G	0.28	0.105	0.017
rs9304665	19	47602577	A	T	0.77	0.103	0.018
rs2115107	19	7968168	A	G	0.38	0.069	0.016
rs116843064	19	8429323	A	G	0.02	-0.150	0.055
rs7554251	1	11317932	C	T	0.73	0.036	0.017
rs1127215	1	117532790	T	C	0.42	-0.065	0.016
rs66464442	1	118171801	A	C	0.32	0.121	0.016
rs1493694	1	120526982	T	C	0.11	0.146	0.025
rs145904381	1	151017991	C	T	0.01	-0.266	0.071
rs2297607	1	154320942	G	A	0.24	0.051	0.018
rs6696888	1	155508882	A	G	0.68	-0.048	0.016
rs7546252	1	172368310	G	A	0.56	-0.092	0.015
rs539515	1	177889025	C	A	0.21	0.049	0.019
rs2816177	1	179248952	G	A	0.41	0.049	0.016
rs41304257	1	201849926	G	A	0.28	-0.042	0.017
rs61817176	1	206621028	C	A	0.52	-0.074	0.015
rs10916780	1	20707153	G	A	0.20	-0.045	0.019

rs340874	1	214159256	C	T	0.57	0.166	0.015
rs1337101	1	219726100	T	G	0.32	-0.095	0.016
rs348330	1	229672955	A	G	0.63	-0.119	0.016
rs10925635	1	235573486	C	A	0.64	0.046	0.016
rs17261915	1	26756856	C	T	0.25	0.074	0.018
rs3753693	1	29060898	T	C	0.41	-0.066	0.016
rs61779284	1	39855177	A	G	0.21	0.130	0.019
rs79090772	1	51209148	C	T	0.09	-0.219	0.027
rs2269247	1	64107284	T	C	0.18	-0.056	0.020
rs11583755	1	6672729	C	A	0.36	0.107	0.016
rs2613499	1	72751552	G	A	0.19	-0.052	0.019
rs10159026	1	96404462	T	C	0.25	-0.062	0.018
rs6137042	20	2100095	A	G	0.20	-0.050	0.019
rs7274134	20	22428284	T	C	0.25	-0.062	0.018
rs6059662	20	32675727	G	A	0.65	0.037	0.016
rs2038457	20	42239145	G	A	0.81	0.041	0.020
rs12625671	20	42994812	C	T	0.11	0.118	0.025
rs6066138	20	45594711	A	G	0.28	-0.135	0.017
rs6021276	20	50155386	C	T	0.64	-0.074	0.016
rs865034	20	51261615	C	T	0.66	0.040	0.016
rs4810145	20	57396495	C	T	0.52	0.068	0.015
rs6011155	20	62450664	C	T	0.37	-0.074	0.016
rs2240716	22	19969696	T	C	0.30	0.074	0.017
rs56392746	22	30451688	A	G	0.09	-0.138	0.026
rs75307421	22	32203334	A	G	0.02	0.151	0.061
rs138771	22	35705359	G	A	0.81	-0.055	0.020
rs1801645	22	50356850	T	C	0.74	-0.059	0.018
rs34506349	2	100598726	A	G	0.04	-0.099	0.038
rs79950062	2	111940612	C	T	0.13	-0.053	0.023
rs9308614	2	121337196	G	A	0.15	-0.090	0.022
rs6716394	2	146350724	A	G	0.54	-0.045	0.015
rs4668483	2	16231732	G	A	0.68	-0.040	0.016

rs10184004	2	165508389	T	C	0.41	-0.115	0.016
rs11680058	2	16574669	A	G	0.87	0.104	0.025
rs13406280	2	166610827	T	C	0.49	-0.047	0.015
rs72917531	2	175238176	A	C	0.19	-0.078	0.020
rs36051007	2	179545859	T	C	0.32	-0.035	0.017
rs67383253	2	181570394	C	T	0.37	-0.035	0.016
rs6712905	2	196952010	C	T	0.26	0.048	0.018
rs4482463	2	205375909	A	C	0.92	-0.063	0.029
rs34329895	2	208870017	G	A	0.60	-0.063	0.016
rs2943650	2	227105921	T	C	0.65	0.143	0.016
rs13415288	2	228971884	C	T	0.34	0.059	0.016
rs34339006	2	234271522	T	C	0.39	0.092	0.016
rs1260326	2	27730940	C	T	0.61	0.156	0.016
rs77165542	2	430975	T	C	0.04	-0.155	0.042
rs921069	2	43206922	G	A	0.58	-0.038	0.016
rs76675804	2	43611883	C	T	0.10	-0.311	0.026
rs10193538	2	58981064	T	G	0.61	0.072	0.016
rs243018	2	60586707	G	C	0.45	0.088	0.016
rs114213622	2	65243284	T	G	0.01	-0.303	0.080
rs10188334	2	653874	T	C	0.17	-0.087	0.020
rs12185610	2	65661468	C	A	0.41	-0.063	0.016
rs4671799	2	67622243	G	A	0.68	-0.036	0.016
rs4832290	2	86707504	C	T	0.77	-0.053	0.018
rs17036126	3	12287863	T	C	0.13	0.127	0.023
rs11708067	3	123065778	G	A	0.25	-0.262	0.018
rs17036160	3	12329783	T	C	0.12	-0.088	0.024
rs9873519	3	124921457	T	C	0.53	0.097	0.015
rs1224997	3	131631201	T	C	0.28	0.072	0.017
rs667920	3	136069472	T	G	0.77	0.038	0.018
rs9289556	3	138033181	T	C	0.73	-0.077	0.017
rs56243018	3	141101839	C	A	0.05	-0.214	0.036
rs28502438	3	149220109	C	T	0.43	-0.051	0.016

rs7633673	3	152084243	A	G	0.41	-0.086	0.016
rs11706810	3	160159921	C	T	0.48	-0.106	0.015
rs13099581	3	168226052	T	C	0.14	-0.060	0.022
rs8192675	3	170724883	C	T	0.29	-0.188	0.017
rs6444036	3	184901216	T	G	0.16	0.041	0.021
rs9859406	3	185534482	A	G	0.31	0.167	0.017
rs2041965	3	186648411	T	C	0.34	-0.083	0.016
rs6777684	3	187741842	G	A	0.61	0.134	0.016
rs13094957	3	23457080	C	T	0.20	-0.131	0.019
rs1470560	3	35670150	A	G	0.37	0.037	0.016
rs2624847	3	50174197	T	G	0.74	-0.084	0.017
rs13434089	3	63948566	C	T	0.12	-0.082	0.024
rs9870517	3	64708600	C	A	0.40	-0.096	0.016
rs1374915	3	71668037	C	T	0.42	-0.036	0.016
rs1523766	3	77670448	G	A	0.50	-0.031	0.015
rs978444	3	93981060	T	G	0.55	-0.057	0.015
rs3872707	3	9514016	A	G	0.12	0.049	0.023
rs7659468	4	103895317	G	T	0.49	-0.103	0.015
rs11728350	4	106078097	G	A	0.13	0.110	0.023
rs77141743	4	121774048	A	G	0.16	0.045	0.021
rs730831	4	1240299	G	T	0.04	-0.123	0.041
rs2604918	4	140879929	T	G	0.33	-0.063	0.016
rs2125799	4	156697784	C	T	0.33	0.060	0.016
rs28819812	4	157652753	A	C	0.32	-0.060	0.016
rs4865436	4	1788130	G	C	0.29	0.050	0.018
rs2169033	4	18044357	T	C	0.68	0.081	0.017
rs55691245	4	185716100	A	G	0.14	-0.160	0.022
rs7664347	4	20265535	C	T	0.64	-0.040	0.016
rs10938398	4	45186139	A	G	0.43	0.040	0.016
rs1996617	4	52798624	C	T	0.29	0.101	0.017
rs114447556	4	53207093	T	C	0.08	0.080	0.029
rs10937721	4	6306763	C	G	0.59	0.142	0.016

rs73222806	4	753840	G	C	0.05	0.098	0.035
rs6835992	4	76496817	G	A	0.69	0.066	0.017
rs993380	4	83584496	G	A	0.67	-0.059	0.016
rs28408270	4	95114572	T	G	0.47	-0.050	0.015
rs1961224	4	95999825	G	A	0.35	-0.065	0.016
rs141146025	5	101966291	A	C	0.02	0.129	0.058
rs75432112	5	102586407	A	G	0.05	0.195	0.036
rs329118	5	133861663	T	C	0.42	0.041	0.016
rs111686785	5	14738965	G	A	0.03	0.182	0.044
rs72734782	5	14789003	G	A	0.21	0.066	0.019
rs12514030	5	14810110	G	T	0.12	-0.103	0.023
rs1650505	5	158029734	A	G	0.21	0.060	0.019
rs4343858	5	176679407	A	G	0.23	-0.042	0.018
rs138373837	5	36219710	T	C	0.02	0.101	0.050
rs62366821	5	44875449	G	A	0.49	-0.055	0.015
rs10067659	5	52084365	C	G	0.79	-0.081	0.019
rs4865796	5	53272664	A	G	0.69	0.049	0.017
rs464605	5	55807370	T	C	0.75	0.080	0.019
rs34341	5	74934009	T	A	0.58	0.073	0.016
rs7732130	5	76435004	A	G	0.68	-0.132	0.016
rs6870983	5	87697533	T	C	0.21	-0.067	0.019
rs34483452	5	87986314	A	C	0.14	0.077	0.023
rs7752666	6	107445266	T	C	0.32	-0.035	0.017
rs80196932	6	117996631	C	T	0.16	-0.064	0.021
rs11759026	6	126792095	G	A	0.23	0.136	0.018
rs2876354	6	137295352	T	C	0.47	-0.083	0.016
rs7742292	6	138864489	C	T	0.40	0.041	0.016
rs2982521	6	139835329	T	A	0.63	-0.110	0.016
rs9390022	6	143056556	C	T	0.38	-0.042	0.016
rs1538247	6	153395344	C	T	0.30	0.093	0.017
rs2179168	6	15477030	A	G	0.80	0.046	0.019
rs501470	6	160770918	G	T	0.47	-0.089	0.015

rs4709746	6	164133001	T	C	0.13	-0.050	0.023
rs7774074	6	20517130	A	C	0.21	0.039	0.019
rs35261542	6	20675792	A	C	0.26	0.268	0.017
rs3117189	6	32033944	G	A	0.85	0.281	0.021
rs2780215	6	34236973	G	A	0.07	-0.110	0.033
rs7748962	6	43759927	A	G	0.77	0.113	0.018
rs9472139	6	43813711	C	G	0.29	0.065	0.017
rs3798519	6	50788778	C	A	0.18	0.107	0.020
rs9370243	6	53789830	T	G	0.08	0.079	0.028
rs9449295	6	64163807	C	T	0.54	0.036	0.015
rs9379084	6	7231843	A	G	0.12	-0.198	0.025
rs187653072	7	102976385	C	T	0.03	0.134	0.044
rs73184014	7	104516274	G	A	0.22	-0.053	0.019
rs6976111	7	117495667	A	C	0.30	0.074	0.017
rs13237518	7	12269593	A	C	0.41	0.048	0.016
rs3996350	7	130427057	C	G	0.50	-0.086	0.015
rs60251368	7	140522073	G	A	0.06	0.096	0.034
rs4252505	7	142607301	G	A	0.06	0.070	0.031
rs17168486	7	14898282	T	C	0.17	0.162	0.020
rs4725959	7	150534741	G	A	0.22	0.042	0.019
rs10228796	7	15064190	G	C	0.55	0.160	0.015
rs6946660	7	156948648	C	T	0.35	-0.107	0.016
rs11762413	7	2090387	G	C	0.25	-0.085	0.018
rs2188848	7	23884697	G	A	0.20	-0.055	0.019
rs860262	7	28194397	A	C	0.50	-0.158	0.015
rs917195	7	30728452	T	C	0.23	-0.073	0.018
rs730497	7	44223721	A	G	0.18	0.445	0.020
rs73121277	7	50577968	C	T	0.28	0.084	0.017
rs6975279	7	69649683	A	C	0.26	0.101	0.018
rs6956980	7	89803634	C	T	0.53	0.083	0.015
rs7834323	8	10671984	C	T	0.29	-0.074	0.017
rs727582	8	116650468	G	A	0.34	-0.093	0.016

rs13266634	8	118184783	T	C	0.31	-0.277	0.017
rs12056338	8	12643055	T	G	0.42	0.050	0.016
rs17772814	8	128711742	A	G	0.08	-0.099	0.029
rs1561927	8	129568078	T	C	0.73	-0.048	0.017
rs35753840	8	14148990	C	A	0.39	0.054	0.016
rs13268508	8	145525277	T	C	0.38	0.087	0.016
rs2953845	8	145972950	T	C	0.55	0.042	0.015
rs6558173	8	22492103	T	G	0.35	0.039	0.016
rs2725370	8	30852826	C	T	0.70	-0.049	0.017
rs57735787	8	34438332	G	A	0.25	-0.042	0.018
rs13262861	8	41508577	A	C	0.17	-0.121	0.021
rs7813865	8	57506937	C	T	0.29	0.041	0.017
rs10101067	8	72407374	C	G	0.08	0.092	0.029
rs28792187	8	74568099	G	A	0.07	0.123	0.030
rs1895874	8	95675372	A	G	0.50	0.048	0.015
rs10808671	8	95967372	G	A	0.53	-0.073	0.015
rs60384372	8	9974584	G	A	0.47	-0.056	0.015
rs1567353	9	1033773	G	C	0.31	0.035	0.017
rs10119430	9	111938268	A	G	0.79	-0.054	0.019
rs1431819	9	116943357	G	A	0.70	0.038	0.017
rs10818763	9	125689694	T	C	0.13	-0.108	0.023
rs10739629	9	126093422	T	C	0.51	-0.036	0.015
rs529565	9	136149500	C	T	0.32	0.164	0.017
rs28642213	9	139248082	G	A	0.75	0.169	0.018
rs12380322	9	19074538	G	A	0.39	0.051	0.016
rs10965247	9	22132729	G	A	0.18	-0.302	0.020
rs7018475	9	22137685	G	T	0.26	0.178	0.018
rs11788619	9	22258082	T	A	0.03	-0.134	0.048
rs2150854	9	28411949	T	G	0.33	0.072	0.016
rs4237150	9	4290085	C	G	0.40	0.091	0.016
rs67269808	9	81907986	G	A	0.06	-0.130	0.032
rs2796441	9	84308948	A	G	0.42	-0.096	0.016

rs7023781	9	96447178	T	C	0.27	0.058	0.017
rs10993072	9	96915002	T	C	0.32	0.083	0.016
rs28496034	9	98278332	G	C	0.33	-0.057	0.016

EAF: effect allele frequency; SE: standard error; SNP: single nucleotide polymorphism.

Table S5. Genetic proxies for glycemic control by any mechanism, and estimates for their association with glycated hemoglobin (for the outcome of left ventricular ejection fraction).

SNP	Chromosome	Position	Effect allele	Other allele	EAF	Beta	SE
rs2482506	10	104563743	G	C	0.25	-0.053	0.018
rs7090695	10	112801213	C	G	0.80	0.060	0.019
rs11196174	10	114734096	G	A	0.29	0.252	0.017
rs4918790	10	114830254	A	G	0.91	-0.117	0.028
rs4752351	10	121685016	C	T	0.20	0.087	0.019
rs11257655	10	12307894	T	C	0.21	0.219	0.019
rs946859	10	13565429	A	G	0.47	-0.075	0.015
rs3122231	10	44027356	C	T	0.65	0.050	0.016
rs949693	10	70354574	A	G	0.61	-0.050	0.016
rs11592899	10	71333783	A	G	0.34	-0.055	0.016
rs2812535	10	71456857	A	G	0.62	0.069	0.016
rs697239	10	80947438	C	T	0.46	-0.105	0.015
rs11201992	10	88117318	A	C	0.46	-0.038	0.015
rs1111875	10	94462882	T	C	0.41	-0.181	0.016
rs66536955	10	94737667	C	T	0.26	0.044	0.017
rs34041345	10	99174580	G	T	0.26	0.060	0.018
rs529623	11	117693255	C	T	0.52	-0.059	0.015
rs10893830	11	128044159	T	C	0.13	-0.058	0.023
rs10750397	11	128234144	G	A	0.72	-0.040	0.017
rs67232546	11	128398938	T	C	0.21	0.067	0.019
rs757110	11	17418477	A	C	0.64	-0.112	0.016
rs11042987	11	2201059	A	C	0.58	-0.034	0.016
rs2283167	11	2580063	A	G	0.14	-0.055	0.023
rs231362	11	2691471	G	A	0.52	0.120	0.015
rs10767659	11	27686196	T	G	0.67	-0.041	0.016
rs60808706	11	2857233	A	G	0.05	-0.227	0.035
rs2289488	11	2892955	C	G	0.40	0.040	0.016

rs74673753	11	32623621	T	A	0.06	-0.106	0.033
rs2956092	11	34908780	C	T	0.69	-0.059	0.017
rs3816605	11	47857253	C	T	0.45	-0.080	0.015
rs7483027	11	58128015	C	T	0.38	-0.061	0.016
rs174541	11	61565908	C	T	0.36	-0.098	0.016
rs12789028	11	65326154	A	G	0.20	0.084	0.019
rs11602873	11	72460762	T	A	0.16	-0.187	0.021
rs11236524	11	75464344	C	T	0.09	0.069	0.027
rs2513505	11	76230357	A	C	0.60	0.033	0.016
rs12802861	11	8387806	T	C	0.28	-0.052	0.017
rs10830963	11	92708710	G	C	0.28	0.297	0.017
rs3020069	11	93057087	A	G	0.68	0.093	0.016
rs1426371	12	108629780	A	G	0.26	-0.074	0.018
rs79310463	12	118406696	T	C	0.13	0.104	0.023
rs56348580	12	121432117	C	G	0.31	-0.037	0.017
rs7975763	12	123604053	T	C	0.20	-0.057	0.019
rs2066827	12	12871099	G	T	0.23	0.102	0.018
rs11614914	12	133070294	T	C	0.33	0.078	0.016
rs12828318	12	133766122	G	A	0.18	-0.057	0.020
rs10841886	12	21864377	C	T	0.23	-0.082	0.018
rs1480029	12	26356032	A	G	0.46	0.042	0.015
rs3751239	12	27963676	G	C	0.20	-0.160	0.019
rs7298690	12	4313438	C	T	0.21	0.060	0.019
rs3217893	12	4403876	T	C	0.09	-0.171	0.030
rs2732469	12	48712932	A	T	0.43	-0.258	0.015
rs61937817	12	57212823	G	T	0.11	0.060	0.024
rs11173646	12	61250814	T	A	0.82	-0.046	0.020
rs2257883	12	66216162	A	G	0.13	0.150	0.023
rs12371967	12	66346714	C	T	0.17	-0.043	0.020
rs10879261	12	71520761	G	T	0.41	0.068	0.016
rs11108094	12	95928113	A	C	0.07	0.099	0.030
rs6538805	12	97849120	C	T	0.39	-0.076	0.016

rs9587811	13	109946882	A	C	0.41	-0.056	0.016
rs314879	13	23309382	T	C	0.79	-0.069	0.019
rs34584161	13	26776999	G	A	0.24	-0.063	0.018
rs380854	13	33574631	A	G	0.58	-0.058	0.016
rs9316500	13	51094114	G	T	0.29	-0.067	0.017
rs7991679	13	58691107	A	T	0.16	-0.081	0.021
rs1215451	13	80715893	A	G	0.29	-0.131	0.017
rs112324411	14	101258584	T	C	0.07	-0.101	0.032
rs2295388	14	101309759	A	G	0.22	-0.073	0.019
rs4906272	14	103376031	T	C	0.16	0.046	0.021
rs12883788	14	33303540	T	C	0.46	0.060	0.015
rs7147483	14	38804675	C	T	0.25	-0.158	0.018
rs723355	14	47304091	A	G	0.50	-0.034	0.015
rs4902002	14	61229411	A	G	0.71	-0.034	0.017
rs242105	14	69459229	C	A	0.28	0.062	0.017
rs7156625	14	79942647	A	G	0.22	0.037	0.019
rs8010382	14	91963722	G	A	0.41	0.046	0.016
rs8043085	15	38828140	T	G	0.23	0.071	0.018
rs11856877	15	40620560	G	A	0.11	0.071	0.024
rs1473781	15	41818917	A	G	0.35	0.067	0.016
rs71472935	15	52565725	C	G	0.11	-0.145	0.025
rs7163757	15	62391608	T	C	0.43	-0.042	0.015
rs7178762	15	63871292	T	C	0.55	-0.057	0.015
rs9479	15	74328576	G	A	0.49	0.052	0.015
rs8033589	15	75596685	A	G	0.76	0.058	0.018
rs12910361	15	77782335	G	A	0.71	0.161	0.017
rs893617	15	90381278	T	C	0.72	-0.136	0.017
rs2290202	15	91512267	T	G	0.13	0.085	0.023
rs9927842	16	15153717	C	T	0.84	-0.056	0.021
rs8056890	16	28897452	A	G	0.36	0.105	0.016
rs8054556	16	29958216	A	G	0.47	0.077	0.015
rs55857387	16	300388	C	T	0.20	-0.142	0.019

rs8061528	16	3656482	T	C	0.21	0.092	0.019
rs2024449	16	53494617	C	T	0.44	-0.056	0.015
rs1421085	16	53800954	C	T	0.40	0.154	0.016
rs56125990	16	69742387	G	A	0.15	0.065	0.021
rs4788815	16	71634811	T	A	0.66	0.056	0.016
rs72802365	16	75246035	C	G	0.08	-0.163	0.029
rs2966117	16	81599271	T	G	0.48	0.059	0.015
rs11117364	16	88132199	G	A	0.68	0.066	0.017
rs9937296	16	88554480	C	T	0.86	0.069	0.023
rs66461358	16	89535257	C	T	0.15	0.060	0.021
rs12934854	16	950028	A	G	0.17	0.043	0.020
rs925095	17	17344653	T	C	0.39	-0.075	0.016
rs2297508	17	17715317	G	C	0.65	-0.150	0.016
rs9913225	17	27570622	A	G	0.58	-0.075	0.016
rs1109442	17	34862220	C	T	0.47	0.071	0.015
rs3110641	17	36047417	G	A	0.78	0.091	0.019
rs11651755	17	36099840	T	C	0.51	-0.124	0.015
rs3786017	17	3830340	C	T	0.11	0.054	0.025
rs8071043	17	3988451	C	T	0.33	0.066	0.016
rs1905339	17	40582296	C	T	0.34	0.097	0.016
rs35895680	17	47060322	A	C	0.33	-0.089	0.016
rs366577	17	4854480	T	C	0.60	-0.051	0.016
rs57767539	17	62203059	A	G	0.07	0.136	0.031
rs11658220	17	65646092	A	G	0.10	0.100	0.025
rs12603589	17	65825248	C	T	0.19	0.103	0.020
rs7224711	17	76772288	T	C	0.53	-0.069	0.015
rs303760	18	21083738	T	C	0.35	0.052	0.016
rs16965062	18	31581247	T	C	0.43	0.034	0.015
rs7227272	18	36746623	A	G	0.10	-0.062	0.026
rs410150	18	40066006	T	C	0.80	-0.047	0.019
rs17596995	18	53166594	A	G	0.20	-0.049	0.019
rs1517037	18	56878274	T	C	0.19	-0.093	0.020

rs6567160	18	57829135	C	T	0.23	0.097	0.018
rs74625348	18	60846430	C	G	0.23	-0.044	0.019
rs12963820	18	63426213	A	T	0.27	0.034	0.017
rs7240767	18	7070642	C	T	0.39	0.063	0.016
rs6565922	18	74558999	T	C	0.38	0.078	0.016
rs9384	19	13010643	T	G	0.38	-0.107	0.016
rs10404726	19	18834514	T	C	0.47	-0.035	0.015
rs58542926	19	19379549	T	C	0.08	0.139	0.029
rs924150	19	31829903	C	A	0.39	-0.087	0.016
rs4805881	19	33896432	C	A	0.67	-0.077	0.016
rs429358	19	45411941	C	T	0.16	-0.142	0.021
rs8107527	19	46158417	A	G	0.28	0.105	0.017
rs9304665	19	47602577	A	T	0.77	0.103	0.018
rs2115107	19	7968168	A	G	0.38	0.069	0.016
rs7554251	1	11317932	C	T	0.73	0.036	0.017
rs1127215	1	117532790	T	C	0.42	-0.065	0.016
rs66464442	1	118171801	A	C	0.32	0.121	0.016
rs1493694	1	120526982	T	C	0.11	0.146	0.025
rs115983556	1	149873582	C	A	0.08	-0.153	0.028
rs1194592	1	154324384	G	C	0.44	-0.044	0.015
rs3020781	1	155269776	G	A	0.27	0.086	0.017
rs7546252	1	172368310	G	A	0.56	-0.092	0.015
rs539515	1	177889025	C	A	0.21	0.049	0.019
rs2816177	1	179248952	G	A	0.41	0.049	0.016
rs41304257	1	201849926	G	A	0.28	-0.042	0.017
rs61817176	1	206621028	C	A	0.52	-0.074	0.015
rs10916780	1	20707153	G	A	0.20	-0.045	0.019
rs340874	1	214159256	C	T	0.57	0.166	0.015
rs1337101	1	219726100	T	G	0.32	-0.095	0.016
rs348330	1	229672955	A	G	0.63	-0.119	0.016
rs10925635	1	235573486	C	A	0.64	0.046	0.016
rs17261915	1	26756856	C	T	0.25	0.074	0.018

rs3753693	1	29060898	T	C	0.41	-0.066	0.016
rs61779284	1	39855177	A	G	0.21	0.130	0.019
rs79090772	1	51209148	C	T	0.09	-0.219	0.027
rs2269247	1	64107284	T	C	0.18	-0.056	0.020
rs11583755	1	6672729	C	A	0.36	0.107	0.016
rs2613499	1	72751552	G	A	0.19	-0.052	0.019
rs10159026	1	96404462	T	C	0.25	-0.062	0.018
rs6137042	20	2100095	A	G	0.20	-0.050	0.019
rs7274134	20	22428284	T	C	0.25	-0.062	0.018
rs6059662	20	32675727	G	A	0.65	0.037	0.016
rs2038457	20	42239145	G	A	0.81	0.041	0.020
rs12625671	20	42994812	C	T	0.11	0.118	0.025
rs6066138	20	45594711	A	G	0.28	-0.135	0.017
rs6021276	20	50155386	C	T	0.64	-0.074	0.016
rs865034	20	51261615	C	T	0.66	0.040	0.016
rs4810145	20	57396495	C	T	0.52	0.068	0.015
rs6011155	20	62450664	C	T	0.37	-0.074	0.016
rs2240716	22	19969696	T	C	0.30	0.074	0.017
rs56392746	22	30451688	A	G	0.09	-0.138	0.026
rs138771	22	35705359	G	A	0.81	-0.055	0.020
rs1801645	22	50356850	T	C	0.74	-0.059	0.018
rs79950062	2	111940612	C	T	0.13	-0.053	0.023
rs9308614	2	121337196	G	A	0.15	-0.090	0.022
rs6716394	2	146350724	A	G	0.54	-0.045	0.015
rs4668483	2	16231732	G	A	0.68	-0.040	0.016
rs10184004	2	165508389	T	C	0.41	-0.115	0.016
rs11680058	2	16574669	A	G	0.87	0.104	0.025
rs13406280	2	166610827	T	C	0.49	-0.047	0.015
rs72917531	2	175238176	A	C	0.19	-0.078	0.020
rs36051007	2	179545859	T	C	0.32	-0.035	0.017
rs67383253	2	181570394	C	T	0.37	-0.035	0.016
rs6712905	2	196952010	C	T	0.26	0.048	0.018

rs4482463	2	205375909	A	C	0.92	-0.063	0.029
rs34329895	2	208870017	G	A	0.60	-0.063	0.016
rs2943650	2	227105921	T	C	0.65	0.143	0.016
rs13415288	2	228971884	C	T	0.34	0.059	0.016
rs34339006	2	234271522	T	C	0.39	0.092	0.016
rs1260326	2	27730940	C	T	0.61	0.156	0.016
rs921069	2	43206922	G	A	0.58	-0.038	0.016
rs76675804	2	43611883	C	T	0.10	-0.311	0.026
rs10193538	2	58981064	T	G	0.61	0.072	0.016
rs243018	2	60586707	G	C	0.45	0.088	0.016
rs10188334	2	653874	T	C	0.17	-0.087	0.020
rs12185610	2	65661468	C	A	0.41	-0.063	0.016
rs4671799	2	67622243	G	A	0.68	-0.036	0.016
rs4832290	2	86707504	C	T	0.77	-0.053	0.018
rs17036126	3	12287863	T	C	0.13	0.127	0.023
rs11708067	3	123065778	G	A	0.25	-0.262	0.018
rs17036160	3	12329783	T	C	0.12	-0.088	0.024
rs9873519	3	124921457	T	C	0.53	0.097	0.015
rs1225004	3	131626991	C	T	0.28	0.071	0.017
rs667920	3	136069472	T	G	0.77	0.038	0.018
rs6766859	3	138055136	T	C	0.63	-0.091	0.016
rs34573045	3	149196752	G	C	0.43	0.049	0.015
rs7633673	3	152084243	A	G	0.41	-0.086	0.016
rs11706810	3	160159921	C	T	0.48	-0.106	0.015
rs13099581	3	168226052	T	C	0.14	-0.060	0.022
rs8192675	3	170724883	C	T	0.29	-0.188	0.017
rs6444036	3	184901216	T	G	0.16	0.041	0.021
rs9859406	3	185534482	A	G	0.31	0.167	0.017
rs2041965	3	186648411	T	C	0.34	-0.083	0.016
rs6777684	3	187741842	G	A	0.61	0.134	0.016
rs13094957	3	23457080	C	T	0.20	-0.131	0.019
rs1470560	3	35670150	A	G	0.37	0.037	0.016

rs2624847	3	50174197	T	G	0.74	-0.084	0.017
rs13434089	3	63948566	C	T	0.12	-0.082	0.024
rs9870517	3	64708600	C	A	0.40	-0.096	0.016
rs1374915	3	71668037	C	T	0.42	-0.036	0.016
rs1523766	3	77670448	G	A	0.50	-0.031	0.015
rs978444	3	93981060	T	G	0.55	-0.057	0.015
rs3872707	3	9514016	A	G	0.12	0.049	0.023
rs7659468	4	103895317	G	T	0.49	-0.103	0.015
rs11728350	4	106078097	G	A	0.13	0.110	0.023
rs77141743	4	121774048	A	G	0.16	0.045	0.021
rs2604918	4	140879929	T	G	0.33	-0.063	0.016
rs2125799	4	156697784	C	T	0.33	0.060	0.016
rs28819812	4	157652753	A	C	0.32	-0.060	0.016
rs4865436	4	1788130	G	C	0.29	0.050	0.018
rs2169033	4	18044357	T	C	0.68	0.081	0.017
rs55691245	4	185716100	A	G	0.14	-0.160	0.022
rs7664347	4	20265535	C	T	0.64	-0.040	0.016
rs10938398	4	45186139	A	G	0.43	0.040	0.016
rs1996617	4	52798624	C	T	0.29	0.101	0.017
rs114447556	4	53207093	T	C	0.08	0.080	0.029
rs10937721	4	6306763	C	G	0.59	0.142	0.016
rs75724417	4	757921	T	C	0.05	0.089	0.035
rs6835992	4	76496817	G	A	0.69	0.066	0.017
rs993380	4	83584496	G	A	0.67	-0.059	0.016
rs28408270	4	95114572	T	G	0.47	-0.050	0.015
rs1961224	4	95999825	G	A	0.35	-0.065	0.016
rs116782923	5	102331465	T	A	0.05	0.197	0.034
rs329118	5	133861663	T	C	0.42	0.041	0.016
rs9312873	5	14777799	G	A	0.10	-0.160	0.026
rs1650505	5	158029734	A	G	0.21	0.060	0.019
rs4343858	5	176679407	A	G	0.23	-0.042	0.018
rs62366821	5	44875449	G	A	0.49	-0.055	0.015

rs10067659	5	52084365	C	G	0.79	-0.081	0.019
rs4865796	5	53272664	A	G	0.69	0.049	0.017
rs464605	5	55807370	T	C	0.75	0.080	0.019
rs34341	5	74934009	T	A	0.58	0.073	0.016
rs7732130	5	76435004	A	G	0.68	-0.132	0.016
rs6870983	5	87697533	T	C	0.21	-0.067	0.019
rs34483452	5	87986314	A	C	0.14	0.077	0.023
rs7752666	6	107445266	T	C	0.32	-0.035	0.017
rs80196932	6	117996631	C	T	0.16	-0.064	0.021
rs11759026	6	126792095	G	A	0.23	0.136	0.018
rs2876354	6	137295352	T	C	0.47	-0.083	0.016
rs7742292	6	138864489	C	T	0.40	0.041	0.016
rs2982521	6	139835329	T	A	0.63	-0.110	0.016
rs9390022	6	143056556	C	T	0.38	-0.042	0.016
rs1538247	6	153395344	C	T	0.30	0.093	0.017
rs2179168	6	15477030	A	G	0.80	0.046	0.019
rs501470	6	160770918	G	T	0.47	-0.089	0.015
rs4709746	6	164133001	T	C	0.13	-0.050	0.023
rs7774074	6	20517130	A	C	0.21	0.039	0.019
rs35261542	6	20675792	A	C	0.26	0.268	0.017
rs3117189	6	32033944	G	A	0.85	0.281	0.021
rs2780215	6	34236973	G	A	0.07	-0.110	0.033
rs7748962	6	43759927	A	G	0.77	0.113	0.018
rs9472139	6	43813711	C	G	0.29	0.065	0.017
rs3798519	6	50788778	C	A	0.18	0.107	0.020
rs9370243	6	53789830	T	G	0.08	0.079	0.028
rs9449295	6	64163807	C	T	0.54	0.036	0.015
rs9379084	6	7231843	A	G	0.12	-0.198	0.025
rs62482399	7	102972707	T	C	0.09	0.087	0.027
rs73184014	7	104516274	G	A	0.22	-0.053	0.019
rs6976111	7	117495667	A	C	0.30	0.074	0.017
rs13237518	7	12269593	A	C	0.41	0.048	0.016

rs3996350	7	130427057	C	G	0.50	-0.086	0.015
rs60251368	7	140522073	G	A	0.06	0.096	0.034
rs4252505	7	142607301	G	A	0.06	0.070	0.031
rs17168486	7	14898282	T	C	0.17	0.162	0.020
rs4725959	7	150534741	G	A	0.22	0.042	0.019
rs10228796	7	15064190	G	C	0.55	0.160	0.015
rs6946660	7	156948648	C	T	0.35	-0.107	0.016
rs11762413	7	2090387	G	C	0.25	-0.085	0.018
rs2188848	7	23884697	G	A	0.20	-0.055	0.019
rs860262	7	28194397	A	C	0.50	-0.158	0.015
rs917195	7	30728452	T	C	0.23	-0.073	0.018
rs730497	7	44223721	A	G	0.18	0.445	0.020
rs73121277	7	50577968	C	T	0.28	0.084	0.017
rs6975279	7	69649683	A	C	0.26	0.101	0.018
rs6956980	7	89803634	C	T	0.53	0.083	0.015
rs7834323	8	10671984	C	T	0.29	-0.074	0.017
rs727582	8	116650468	G	A	0.34	-0.093	0.016
rs13266634	8	118184783	T	C	0.31	-0.277	0.017
rs12056338	8	12643055	T	G	0.42	0.050	0.016
rs17772814	8	128711742	A	G	0.08	-0.099	0.029
rs1561927	8	129568078	T	C	0.73	-0.048	0.017
rs35753840	8	14148990	C	A	0.39	0.054	0.016
rs13268508	8	145525277	T	C	0.38	0.087	0.016
rs2953845	8	145972950	T	C	0.55	0.042	0.015
rs6558173	8	22492103	T	G	0.35	0.039	0.016
rs2725370	8	30852826	C	T	0.70	-0.049	0.017
rs57735787	8	34438332	G	A	0.25	-0.042	0.018
rs13262861	8	41508577	A	C	0.17	-0.121	0.021
rs7813865	8	57506937	C	T	0.29	0.041	0.017
rs10101067	8	72407374	C	G	0.08	0.092	0.029
rs28792187	8	74568099	G	A	0.07	0.123	0.030
rs1895874	8	95675372	A	G	0.50	0.048	0.015

rs10808671	8	95967372	G	A	0.53	-0.073	0.015
rs60384372	8	9974584	G	A	0.47	-0.056	0.015
rs1567353	9	1033773	G	C	0.31	0.035	0.017
rs10119430	9	111938268	A	G	0.79	-0.054	0.019
rs1431819	9	116943357	G	A	0.70	0.038	0.017
rs10818763	9	125689694	T	C	0.13	-0.108	0.023
rs10739629	9	126093422	T	C	0.51	-0.036	0.015
rs529565	9	136149500	C	T	0.32	0.164	0.017
rs28642213	9	139248082	G	A	0.75	0.169	0.018
rs12380322	9	19074538	G	A	0.39	0.051	0.016
rs10965247	9	22132729	G	A	0.18	-0.302	0.020
rs7018475	9	22137685	G	T	0.26	0.178	0.018
rs2150854	9	28411949	T	G	0.33	0.072	0.016
rs4237150	9	4290085	C	G	0.40	0.091	0.016
rs67269808	9	81907986	G	A	0.06	-0.130	0.032
rs2796441	9	84308948	A	G	0.42	-0.096	0.016
rs7023781	9	96447178	T	C	0.27	0.058	0.017
rs10993072	9	96915002	T	C	0.32	0.083	0.016
rs28496034	9	98278332	G	C	0.33	-0.057	0.016

EAF: effect allele frequency; SE: standard error; SNP: single nucleotide polymorphism.

Table S6. **Weighted median sensitivity analyses for the association of genetically proxied glucagon-like peptide receptor (GLP1R) agonism and glycemic control more generally with heart failure (HF; 47,309 cases / 930,014 controls) and left ventricular ejection fraction (LVEF; *n*=16,923).**

**Exposure**	**Outcome**	**N SNPs**	**Effect units**	**Effect**	**P value**
GLP1R	HF	3	Odds ratio	0.77 [0.62, 0.96]	0.02
Glycemia	HF	350	Odds ratio	0.98 [0.96, 1.00]	0.04
GLP1R	LVEF	3	SD change in LVEF	0.18 [-0.07, 0.42]	0.16
Glycemia	LVEF	334	SD change in LVEF	0.00 [-0.02, 0.02]	0.89

SD: standard deviation; SNP: single-nucleotide polymorphism.

Figure S1. Scatter plot displaying genetic associations of the GLP1R genetic proxies with glycated hemoglobin (mmol/mol, x-axis) and heart failure risk (log-odds, y-axis).

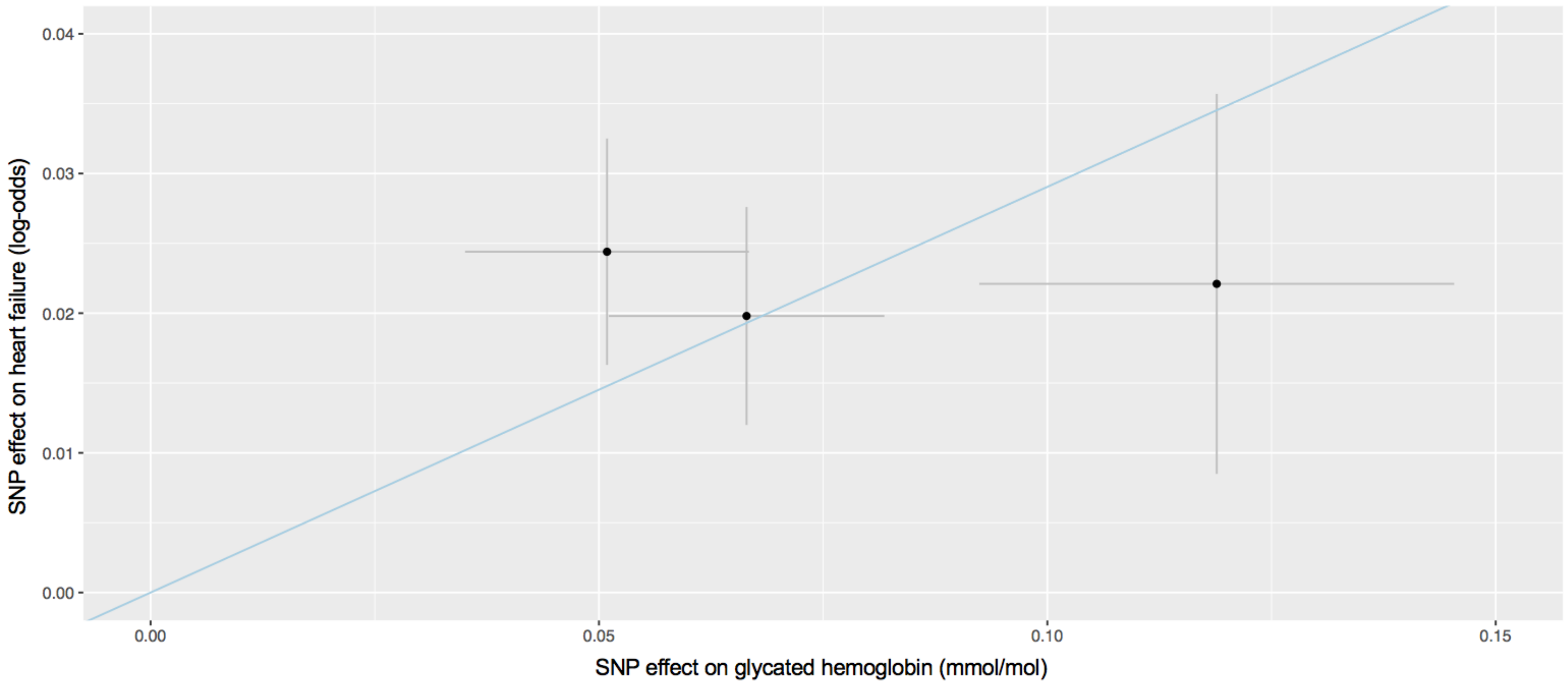

Each point represents a single genetic variant, with vertical and horizontal lines representing standard errors. The slope of the blue diagonal line represents the inverse-variance weighted Mendelian randomization estimate. The *P* value for the Cochran *Q* test for heterogeneity was 0.32. SNP: single-nucleotide polymorphism.

Figure S2. Scatter plot displaying genetic associations of the GLP1R genetic proxies with type 2 diabetes liability (log-odds, x-axis) and heart failure risk (log-odds, y-axis).

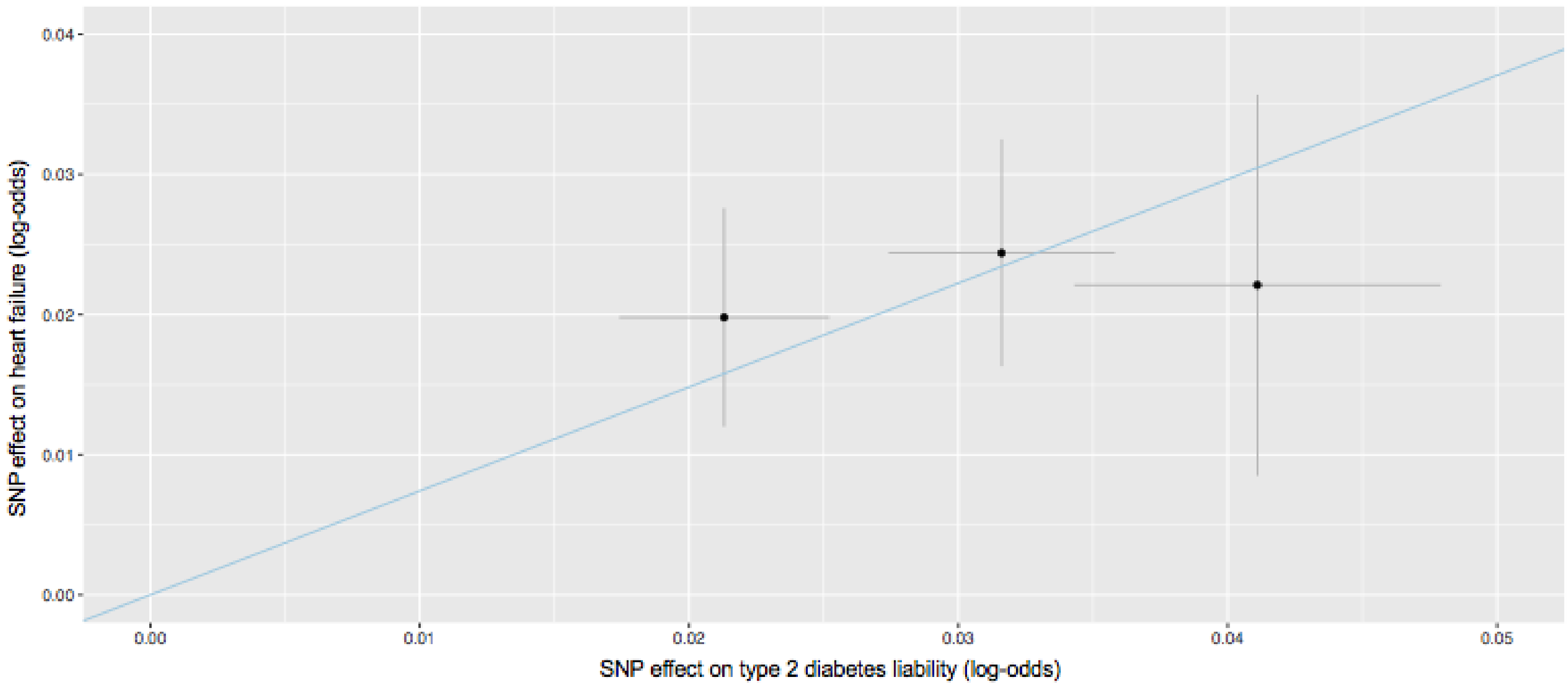

Each point represents a single genetic variant, with vertical and horizontal lines representing standard errors. The slope of the blue diagonal line represents the inverse-variance weighted Mendelian randomization estimate. The *P* value for the Cochran *Q* test for heterogeneity was 0.72. SNP: single-nucleotide polymorphism.

Figure S3. Scatter plot displaying genetic associations of the GLP1R genetic proxies with glycated hemoglobin (mmol/mol, x-axis) and left ventricular ejection fraction (standard deviation units, y-axis).

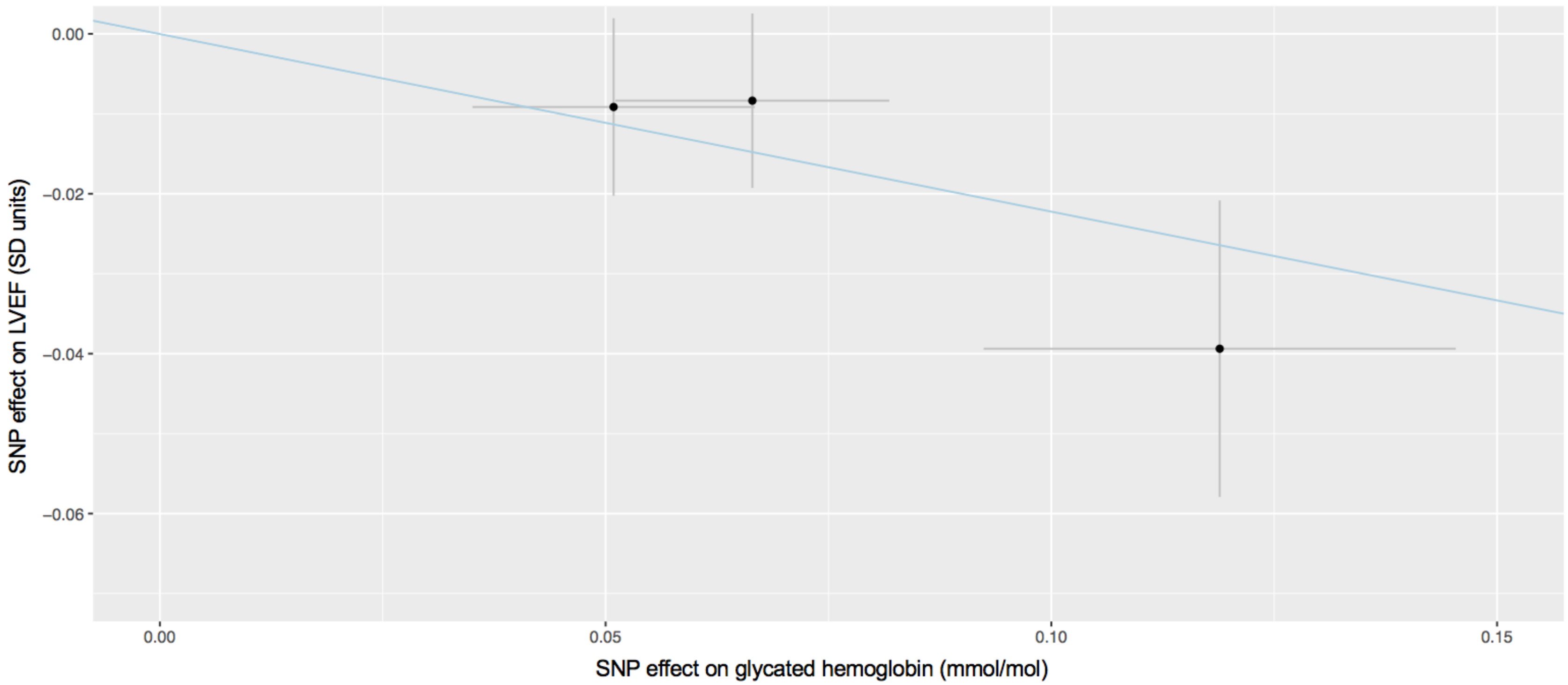

Each point represents a single genetic variant, with vertical and horizontal lines representing standard errors. The slope of the blue diagonal line represents the inverse-variance weighted Mendelian randomization estimate. The *P* value for the Cochran *Q* test for heterogeneity was 0.65. SNP: single-nucleotide polymorphism.

Figure S4. Scatter plot displaying genetic associations of the glycemia genetic proxies with glycated hemoglobin (mmol/mol, x-axis) and heart failure risk (log-odds, y-axis).

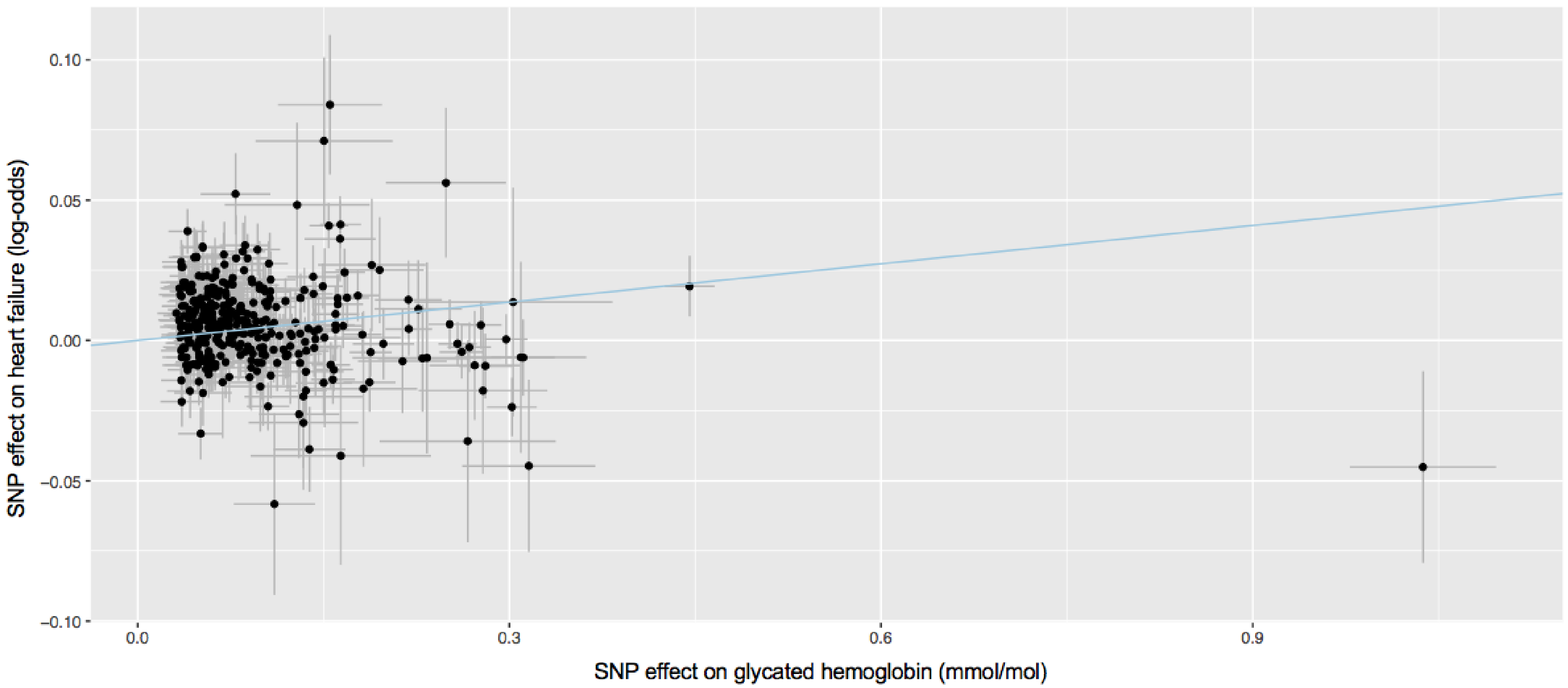

Each point represents a single genetic variant, with vertical and horizontal lines representing standard errors. The slope of the blue diagonal line represents the inverse-variance weighted Mendelian randomization estimate. SNP: single-nucleotide polymorphism.

Figure S5. Scatter plot displaying genetic associations of the glycemia genetic proxies with glycated hemoglobin (mmol/mol, x-axis) and left ventricular ejection fraction (standard deviation units, y-axis).

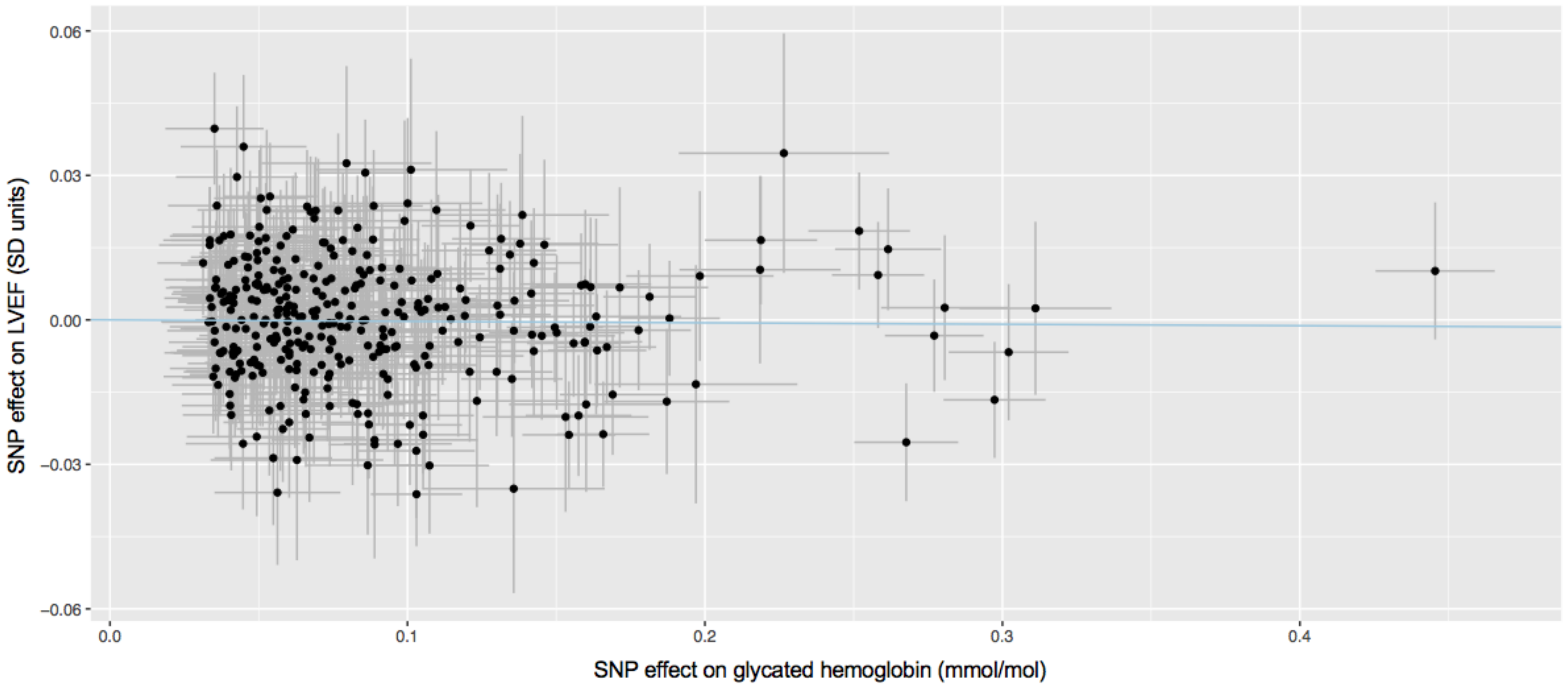

Each point represents a single genetic variant, with vertical and horizontal lines representing standard errors. The slope of the blue diagonal line represents the inverse-variance weighted Mendelian randomization estimate. SNP: single-nucleotide polymorphism.
